# Level of asthma control and risk factors for poor asthma control among clinic patients seen at a Referral Hospital in Addis Ababa, Ethiopia

**DOI:** 10.1186/s13104-017-2887-z

**Published:** 2017-11-06

**Authors:** Tewodros H. Gebremariam, Amsalu B. Binegdie, Abebe S. Mitiku, Aschalew W. Ashagrie, Kibrom G. Gebrehiwot, Dawit K. Huluka, Charles B. Sherman, Neil W. Schluger

**Affiliations:** 1 College of Heath Sciences, Addis Ababa University, Nifas Silk Lafto Subcity, Jemo 1, P O Box 22787, 1000 Addis Ababa, Ethiopia; 2 Alpert Medical School of Brown University, Providence, RI USA; 3 Division of Pulmonary, Allergy and Critical Care Medicine, Columbia University, College of Physicians and Surgeons, New York, NY USA

**Keywords:** Physician-diagnosed asthma, GINA (global initiative for asthma), Risk factors for asthma control

## Abstract

**Objective:**

Uncontrolled asthma negatively impacts patients, families, and the community. The level of symptom control among asthmatics in Ethiopia has not been well studied. We investigated the level of asthma control and risk factors for poor asthma control in clinic patients seen in the largest public hospital in Ethiopia.

**Results:**

In this cross-sectional study, we studied all 182 consecutive subjects with a physician diagnosis of asthma who were seen in chest clinic at Tikur Anbessa Specialized Hospital between July and December 2015. Of the 182 subjects, 68.1% were female. The mean age was 52 ± 12 years and the median duration of asthma was 20 ± 12.7 years. One hundred and seventeen subjects (64.3%) had nighttime awakening due to asthma. Fifty-eight (31%) were not using controller medications and 62 (34.6%) had improper inhaler technique. Only 44 (24.2%) subjects had well-controlled asthma. On multivariate analysis, variables associated with uncontrolled asthma included: use of biomass fuel for cooking, longer duration of asthma (> 30 year), incorrect inhalation technique, and asthma exacerbation in the last 12 months. Most asthmatics attending in the largest public hospital in Ethiopia, had uncontrolled asthma. Several risk factors for poor asthma control were identified. Improved asthma control is possible through directed interventions.

## Introduction

Globally, asthma is recognized as a highly prevalent health problem affecting approximately 300 million people [1] and causing 250,000 premature deaths each year [2]. The number of patients diagnosed with asthma in Ethiopia, Africa’s second most populous country, has been assessed at 2 million people (2.3% of the population) [3]. However, beyond population-based estimates of disease prevalence, little is known about these asthmatics.

Asthma is a major public health problem that negatively impacts patients, their families, and the community by inducing work and school loss, a poor quality of life, frequent emergency visits, hospitalizations, and death [4, 5]. Furthermore, a large proportion of direct and indirect asthma costs have been attributed to severe and uncontrolled asthma [6]. Identifying risk factor for poor asthma control may greatly modify these adverse effects and potentially lessen the financial burden of the disease. The level of asthma control and risk factors for poor asthma control are relatively unknown in Ethiopia.

Numerous studies have been published on asthma control throughout the world and almost all show control to be suboptimal [7–20]. Three small studies investigating asthma control in hospital chest clinic patients in Ethiopia found control to be poor, ranging from 43.8 to 76.1% uncontrolled disease [21–23].

This study was designed to study the level of asthma control and risk factors for poor asthma control in the chest clinic of the largest public hospital in the capital city of Addis Ababa, Ethiopia.

## Main text

### Methods

In this cross sectional study, all consecutive subjects with a physician diagnosis of asthma seen in chest clinic at Tikur Anbessa Specialized Hospital (TASH) in Addis Ababa, between July and December 2015, were included. TASH is the largest tertiary hospital in Ethiopia, offering diagnosis and treatment for approximately 370,000–400,000 patients per year. There are 16 outpatient clinics located within the hospital. Chest clinic has over 500 visits/month; asthma patients account for nearly one-third of these monthly visits. This setting was chosen because of the number of asthmatics and the well-organized longitudinal database.

All adult patients with physician diagnosed asthma seen during the study period were recruited. Subjects were enrolled if they fulfilled the following inclusion criteria: asthma medications use for at least the previous 6 months and 18 + years of age. Those with active lung infections, physician diagnosed bronchiectasis or chronic obstructive pulmonary disease (COPD), or incomplete data were excluded from the study.

Demographics, respiratory symptoms (i.e., cough, breathlessness, wheeze, and chest tightness), current medications, comorbidities, and potentially modifiable risk factors for poor asthma control were obtained from clinic records. Asthma exacerbation was defined as the self-report of worsening respiratory symptoms for greater than 48 h in the past 12 months. GERD was recorded as the self-report of heartburn requiring medication and allergic rhinitis as the self-report of recurrent episodes of running nose and sneezing unrelated to cold symptoms.

Lung function was measured using a Diagnostic EasyOne Plus model 2001 SN spirometer, which was calibrated according to the manufacturer’s recommendations. Spirometric acceptability and reproducibility were determined using the published criteria of the European Respiratory Society and the American Thoracic Society [24].

Asthma severity and control were assessed based on the GINA asthma symptom control assessment tool [25], which has been correlated to other standardized asthma control scores [26–28].

This tool uses frequency of symptoms, night waking due to asthma, limitation of activity, and frequency of reliever medication use. Accordingly, “well controlled” asthma was defined by the absence of daytime symptoms (no more than twice a week), the absence of nighttime symptoms, no limitations in activities, and limited need for rescue medication (not more than twice a week). “Partially controlled” asthma was present when daytime symptoms or rescue medication use was present more than twice per week, and night waking or activity limitation were present in any week, while “uncontrolled” asthma was defined as the presence of any three or more of these individual features within any week [25].

Statistical analysis was performed using IBM SPSS statistics Version 20 (Armonk, NY: IBM Corp). Categorical variables were summarized as frequencies and percentages while continuous data were described using mean, median, standard deviation, or interquartile range. While studying the factors associated with asthma control, uncontrolled asthma was compared to controlled asthma (well and partially controlled). The association between potential factors and uncontrolled asthma was explored using univariate logistic regression. All the factors that showed a p ≤ 0.20 were assessed in a multivariate logistic regression model using a stepwise strategy to identify independent factors associated with poorly controlled asthma. Odds ratios (OR) and their 95% confidence intervals (CI) were determined. A p < 0.05 was regarded as statistically significant.

The institutional review board approved the study protocol and all subjects signed informed consent.

### Results

Baseline subject characteristics are shown in Table 1. One hundred eighty-eight subjects were recruited; 6 were excluded due to incomplete data. Of the 182 study subjects, 68.1% were female. The mean age was 52 ± 12 years and the median duration of asthma was 20 ± 12.7 years. Thirteen (7.3%) were ever smokers. One hundred sixty-five (90.6%) were from urban area; 66 (37.7%) used biomass fuel for cooking. Fifty (27.4%) were unable to read or write. Only 13.6% (9/66 of subjects with recorded weights) had body mass index (BMI) ≥ 30.

**Table 1:** Baseline characteristics of physician diagnosed asthmatics clinic patients seen at a Referral Hospital in Addis Ababa, Ethiopia (n = 182)

Variable	Characteristic	Frequency	Percent
Age (year): mean ± SD		52 ± 12	
Gender	Male	58	31.9
	Female	124	68.1
Duration of asthma, median, years		20 ± 12.7	
School education	≤ Primary level	95	52.5
	≥ Secondary level	86	47.5
Smoking status	Current smoker^a^	2	1.1
	Ex-smoker	11	6.2
	Non smoker	164	90.7
Use of biomass fuel for cooking	Yes	66	37.7
	No	109	62.3
Body mass index^b^	Normal/under weight	37	56.1
	Overweight	20	30.3
	Obese	9	13.6
Exacerbation in the last 12 month	Yes	93	51.1
	No	89	48.9
Allergic rhinitis	Yes	11	6
	No	171	94
GERD	Yes	17	9.3
	No	165	90.7
Incorrect inhalation technique	Yes	62	34.6
	No	117	65.4
Controller medication	Yes	124	68.1
	No	58	31.9
FEV1	> 80%	24	25
	< 80%	72	75
GINA symptom assessment	Well controlled	44	24.2
	Partially controlled	41	22.5
	Uncontrolled	97	53.3

^a^Current smoker = person who was smoking during the time of study

^b^Normal BMI = 18–25 M^2^/kg

One hundred and five subjects (57.7%) had daytime symptoms (wheeze or dyspnea) more than twice per week, 117 (64.3%) had nighttime awakening, and 52 (28.6%) had activity limitations. Ninety-three (51.1%) had experienced an asthma exacerbation in the past 12 months.

One hundred and five subjects (57.7%) used inhaled corticosteroids (ICS) but only 95 (52.2%) consistently. Nineteen (10.4%) used a combination ICS and long acting beta agonist (LABA), and 173 (95.1%) used an inhaled short acting beta agonist (SABA) (salbutamol). Oral steroids were used by 25 (13.7%) of the group. Fifty-eight (31%) were not using controller medications; 62 (34.6%) demonstrated improper inhaler technique.

Spirometry was performed in only 96 (52.7%) subjects due to difficulties in scheduling. Of the ninety-six subjects with spirometry, FEV1/FVC was < 70% in 73 (76% of the group). FEV1 was < 60% in 43 (58.9%), 60–80% in 29 (39.7%), and > 80% in 1 (1.4%).

Only 44 (24.2%) subjects had well-controlled asthma; 41 (22.5%) were partially controlled and 97 (53.3%) were uncontrolled. Among the risk factors studied for uncontrolled asthma, incorrect inhaler technique, duration of asthma, asthma exacerbation in the past 12 months, use of biomass fuel, and excess salbutamol use (> 200 doses/month) were significant.

In the multivariate analysis (Table 2), longer duration of asthma (> 30 year) (Adjusted OR 3.0, 95% CI 1.07–8.3, p = 0.035), incorrect inhaler technique (Adjusted OR 2.5, 95% CI 1.26–4.98, p = 0.008), asthma exacerbation in the last 12 months (Adjusted OR 2.41, 95% CI 1.28–4.54, p = 0.006), and use of biomass fuel for cooking (Adjusted OR 1.99, 95% CI 1.05–3.7, p = 0.034) were found to be associated with uncontrolled asthma.

**Table 2:** Multivariate analysis of factors associated with asthma control (N = 182)

Variable	Character	Level of asthma control			Crude OR (95% CI)	Adjusted OR (95% CI)
		Controlled	Uncontrolled	p value		
Duration of asthma	< 10 years	28 (50.9%)	27 (49.1%)			
	10–20 years	25 (44.6%)	31 (55.4%)			
	21–30 years	24 (55.8%)	19 (44.2%)	0.035	3 (1.08–8.33)	3 (1.07–8.3)
	> 30 years	8 (29.6%)	19 (70.4%)			
Use of biomass fuel	Yes	142 (36.4%)	42 (63.6%)		1.99 (1.063–3.725)	1.99 (1.05–3.7)
	No	58 (53.2%)	51 (46.8%)	0.034		
Exacerbation in last 12 month	Yes	33 (42.1%)	60 (64.5%)	0.006	2.5 (1.4–4.6)	2.41 (1.28–4.54)
	No	45 (51.7%)	37 (41.6%)			
Incorrect inhalation technique	Yes	18 (29%)	44 (71.5%)	0.008	3.05 (1.58–5.9)	2.5 (1.26–4.98)
	No	65 (55.6%)	52 (44.4%)			
Salbutamol use	Yes	77 (44.5%)	96 (55.5%)	0.096	8.7 (1.051–72.4)	6.2 (0.724–54.87)
	No	7 (87.5%)	1 (12.5%)			

### Discussion

We present the first detailed examination of lung function, symptoms, and disease control among a cohort of physician diagnosed asthmatics seen in chest clinic at TASH in Addis Ababa, the largest hospital in Ethiopia. We found poor asthma control overall and significantly impaired lung function in the cohort as a whole.

Numerous studies in North America, Europe, Asia, and the Middle East have almost universally shown asthma control to be suboptimal [7–20]. However, asthma control in Africa has not been well studied. In a recent study from Cameroun, 42% of the study population had inadequately controlled asthma [29]. Similar studies from Nigeria, the Maghreb countries, and Ethiopia have reported uncontrolled disease in 82.9, 71.3, and 71.4% of asthmatics, respectively [21, 30, 31]. When we combine the number of asthmatics with partially controlled and uncontrolled disease (138, 85.2%), our results are equivalent to the findings of these latter studies.

This high percent of uncontrolled asthmatics may underestimate the magnitude of the problem in primary care settings. Our patients were recruited from a chest clinic located in a tertiary center. It has previously been demonstrated that asthma patients seen by specialists are more likely to be better managed than those followed by non-specialists [32, 33].

We identified several factors associated with uncontrolled asthma. Incorrect inhaler technique and duration of asthma were the greatest observed risk factors. Inhaler misuse has been associated with increased risk of hospitalization, emergency room visits, oral steroids and antimicrobial use, and poor asthma control as determined by the Asthma Control Test score [34, 35]. Asthma duration has previously been found to be associated with lower lung function, greater methacholine responsiveness, more asthma symptomatology, and greater use of as-needed albuterol, which are all measures of asthma severity [36].

Asthma exacerbation in the previous 12 months was a 2.5-fold risk for uncontrolled asthma. A previous study demonstrated that patients with a recent exacerbation were at increased risk of future exacerbations (odds ratio = 6.33; 95% CI 4.57, 8.76), even after adjustment for demographics and asthma severity [37].

Finally, biomass fuel for cooking was associated with uncontrolled asthma. Some studies have supported this finding while others have refuted such an association [38–44]. Our data add to the evidence that household air pollution from biomass fuel burning is in fact associated with poor disease control.

Use of excess salbutamol inhaler was associated with poor asthma control in this study on bivariate analysis but not on multivariate analysis. Higher mean daily salbutamol use has been associated with future poor asthma control (OR 1.13; 1.02–1.26) and future severe exacerbations in a previously published randomized controlled trial [45].

Age, gender, level of education, use of asthma controller therapy, treatment adherence, comorbid conditions (i.e., gastroesophageal reflux and allergic rhinitis), and lung function tests were not related to asthma control. Similar results have been found in Uganda [46].

Additional studies done in Africa, Asia, and Europe [47–50] have identified risk factors for poor asthma control. In summary, these investigations have identified lower education level, unemployment, heartburn, chronic sinusitis, female gender, reduced FEV1, and recent exacerbations as risk factors for uncontrolled disease.

There are several important implications of our findings. First, patients frequently do not use their inhalers properly. This has been previously reported in various regions of the world, but in very-low resource settings with few physicians in general and even fewer specialists, this highlights the need for intensified and creative efforts at patient education. Second, we add to the accumulating evidence base regarding adverse health effects of biomass fuel burning; this underscores the importance of efforts to develop cleaner fuels that are affordable and available. Third, we demonstrate that patients with long-standing asthma had more symptoms and worse lung function, thus emphasizing the need for prompt recognition and intensive management. Fourth, we think that this study demonstrates the value of specialist expertise in the assessment of the burden and severity of lung disease in resource-limited settings. We feel our findings are likely to be broadly generalizable because of typical risk factors and exposures in the region.

The extent of non-communicable and chronic lung diseases such as asthma in Africa is enormous. Most asthmatics attending chest clinic at our hospital in Ethiopia had uncontrolled or only partially controlled asthma. Several risk factors for poor asthma control were identified. Improved asthma control is possible through directed interventions including improved medication inhalational technique, minimization of biomass fuel exposure, and better control of disease exacerbations. Our findings highlight the magnitude and burden of chronic lung disease in Africa, and demonstrate the great need for attention to their causes and proper treatment.

Strengths of our study include the use of spirometry in assessment of over half the cohort, lack of confounding by cigarette smoking, and examination of patients by qualified pulmonary physicians trained through the East Africa Training Initiative [51]. Our findings point to several areas where efforts could be targeted to improve asthma symptoms and control among patients.

### Limitations

We acknowledge that a limitation of our study may be the reliance on physician diagnosed asthma, and the availability of spirometric results in just over half of the patients in the cohort. A physician diagnosis of asthma has been used successful in other research of this type; however a recent study [52] found that among adults with physician-diagnosed asthma, a current diagnosis could not be confirmed in 33.1% of study subjects, A low prevalence of tobacco use would ordinary give one confidence that patients were in fact more likely to have had asthma. And the episodic nature of symptoms that our patients reported gives us confidence that the physician diagnosis was accurate.

## Abbreviations

AAU: Addis Ababa University
BMI: body mass index
COPD: chronic obstructive pulmonary disease
FEV1: forced expiratory volume in 1 s
FVC: forced vital capacity
GERD: gastroesophageal reflux disease
GINA: global initiative for asthma
ICS: inhaled corticosteroid
LABA: long acting beta agonist
SABA: short acting beta agonist
SPSS: Statistical Package for the Social Sciences
TASH: Tikur Anbessa Specialized Hospital
